# Microwave versus conventional sintering on mechanical properties of 3Y-TZP - A systematic review

**DOI:** 10.6026/9732063002001086

**Published:** 2024-09-30

**Authors:** Padmapriya Puppala, Gaurang Mistry, Vidhi Desai, Rajeev Singh, Mayuri Bachhav, Sanpreet Singh Sachdev

**Affiliations:** 1 Department of Prosthodontics, D.Y. Patil Deemed to be University, School of Dentistry, Navi, Mumbai, Maharashtra, India; 2 Department of Oral Pathology and Microbiology, Bharati Vidyapeeth (Deemed to be University) Dental College and Hospital, Navi Mumbai, Maharashtra, India

**Keywords:** zirconia, microwave sintering, conventional sintering, mechanical properties

## Abstract

This systematic review evaluates the mechanical properties of yttria-stabilized zirconia (3Y-TZP) processed by microwave sintering compared to conventional sintering methods. Zirconia, known for its excellent strength and esthetics, has seen advancements in sintering techniques to enhance its properties. Conventional sintering, while effective, is time-consuming and less energy-efficient. In contrast, microwave sintering, introduced in 1999, offers rapid heating and improved control over temperature and shrinkage. This review, adhering to PRISMA guidelines, included studies from 2000 to 2023 that compared the effects of both sintering methods on relative density, flexural strength, Young's modulus and hardness of zirconia. Results indicate that microwave sintering improves hardness and reduces processing time, whereas conventional sintering provides higher relative density, flexural strength, and Young's modulus. The findings suggest that the choice of sintering technique should align with specific material property requirements, with each method offering distinct advantages for zirconia applications.

## Background:

In the past 75 years, ceramics have significantly impacted Prosthodontics due to their chemical inertness, strength, and excellent esthetics [1]. However, ceramics also exhibit limitations such as brittleness, low tensile strength and high cost [2]. To address these issues, zirconia was introduced in the 1990s for dental prostheses. It has gained prominence due to its exceptional properties, including high mechanical strength, wear resistance, biocompatibility and corrosion resistance at high temperatures [3]. Yttria-stabilized tetragonal zirconia polycrystalline (3Y-TZP) is particularly valued for its aesthetics and adequate strength, making it suitable for a wide range of applications from single-unit crowns to multi-unit bridges, implants, and implant abutments [4]. Zirconia restorations are manufactured using CAD/CAM technology, which involves scanning, designing, milling, and sintering [5]. Sintering, a crucial step in the manufacturing process, involves heating zirconia powder to high temperatures to bond particles into a dense, solid ceramic structure [6]. Fully sintered zirconia is denser and exhibits superior mechanical properties compared to pre-sintered zirconia [7]. Conventional sintering involves heating zirconia powder in a furnace over several hours, transferring heat through conduction, radiation, and convection [8]. While effective, this method has drawbacks such as longer processing times, slower heating rates, and potential non-uniform temperature distribution [9]. To overcome these limitations, microwave sintering was introduced in 1999. This method uses electromagnetic radiation to heat materials internally, resulting in faster sintering times, reduced energy consumption, and better control over shrinkage and temperature [10]. Microwave sintering offers several advantages over conventional sintering, including speed, energy efficiency, precise temperature control and the potential for improved material properties [11]. However, it also faces challenges like the need for specialized equipment and potential issues with uniformity in large-scale production [12]. Recent advancements in sintering methods, such as Spark Plasma Sintering (SPS) and flash sintering, have further expanded the possibilities for zirconia processing, enhancing material properties and efficiency [13, 14]. Considering these advancements, conventional and microwave sintering methods remain prevalent in zirconia restoration manufacturing. This study aims to investigate the efficacy of microwave sintering compared to conventional sintering regarding the flexural strength, relative density, Young's modulus, and hardness of yttria-stabilized zirconia.

## Materials and Methods:

## Review methods:

## Protocol and registration:

The present systematic review was conducted in accordance with the Preferred Reporting Items for Systematic Reviews and Meta-Analyses (PRISMA) guidelines and the protocol was registered at PROSPERO under registration code CRD42023471421.

## Eligibility criteria:

## Inclusion criteria:

[1] Population: Studies including specimens or fabricated tooth crowns made up of monolithic yttria-stabilized zirconia (YSZ).

[2] Intervention: Studies using microwave or speed sintering of YSZ material.

[3] Comparison: Studies using conventional sintering of YSZ material.

[4] Outcome: Studies providing information on mechanical properties of YSZ after sintering, such as flexural strength, fracture toughness, hardness and Weibull modulus.

[5] Study Design: Studies published in English between January 1, 2000 and June 31, 2023, including all types of studies except for case reports/series and review articles. Only full-text articles were included.

## Exclusion Criteria:

[1] Single intervention studies without a comparative group.

[2] Observational studies, review reports, case series, *in-vitro* and animal studies.

[3] Studies providing only abstracts without full text.

[4] Studies in languages other than English.

## Focused review question:

Is there a difference in the efficacy of microwave sintering compared to conventional sintering regarding mechanical properties such as flexural strength, fracture toughness, hardness and Weibull modulus of yttria-stabilized zirconia?

## Search strategy:

Studies were selected based on the PICOS criteria in the review protocol. Two reviewers independently assessed titles and abstracts to identify potentially eligible studies, with any discrepancies discussed with a third reviewer. The primary outcomes measured were mechanical properties of YSZ. The PRISMA guidelines were followed for conducting the meta-analysis. Electronic databases searched included the Cochrane Central Register of Controlled Trials (CENTRAL), MEDLINE, CINAHL, EMBASE, PsycINFO, Scopus and ScienceDirect using controlled vocabulary and free text terms. Articles published from January 1, 2000, to June 31, 2023, were included. Keywords and MeSH terms were used in combination with Boolean operators in advanced search options.

## Selection of studies:

**Figure 1:**
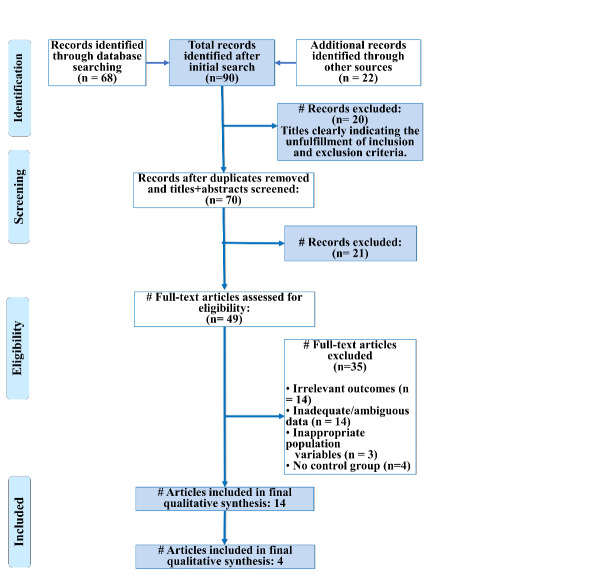
PRISMA flow diagram

Titles and abstracts were reviewed and critically assessed by two independent reviewers. Duplicate records were removed using RevMan software. The screening process of the articles included in the review is explained in the form of the PRISMA flowchart (Figure 1).

The level of concordance between reviewers, calculated through Cohen's kappa, was 0.92 for titles and abstracts and 0.90 for full texts. Discrepancies were resolved by a third reviewer (XYZ) through discussion.

## Data extraction:

Two reviewers independently extracted data from the included studies. Disagreements were resolved through discussion. Data were gathered using a verification list of items, including: Authors, year, and title of the study, country, study design, sample size, age group of participants, gender, intervention, type and volume of YSZ material, comparators, outcomes, methods of outcome assessment, conclusions, and other relevant items. Data for all primary outcomes were recorded in Excel sheets.

## Results:

## Narrative synthesis:

Fourteen studies [15, 16, 16, 17, 18, 19, 19, 20, 21, 22, 23, 24, 25, 26, 26, 27-28] were included in this systematic review whose general characteristics are mentioned in Table 1. All the studies were conducted *in-vitro*. These studies were conducted in different parts of world, with China, Spain, USA, Germany, Turkey, Belgium and Egypt. A total of 720 specimens of zirconia were evaluated in this review of which 360 were speed sintered and remaining was conventionally sintered. Mechanical properties of Zirconia post sintering such as flexural strength, density, and fracture toughness were evaluated. The conclusions of all studies implied that microwave sintering improves the mechanical properties of YSZ, with time and energy consumption reduction.

## Quality assessment of included studies:

Among the included studies, one showed medium risk while the remaining studies showed low risk of bias. In the study by Ai 2015, details of sample size were not mentioned hence the total score of this study was higher as compared to other studies (Table 2).

## Meta-analysis:

Meta-analysis was conducted on studies providing data on similar outcomes irrespective of the type of zirconia material used in the studies.

## Relative density:

**Figure 2:**
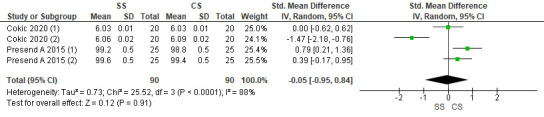
Forest plot for relative density

Two studies were included in the assessment. The pooled value obtained was -0.05[-0.95, 0.84] indicating that the density values were less with speed sintered zirconia as compared to conventionally sintered. Overall, the results were not statistically significant (p>0.05), with 88% heterogeneity. Due to high heterogeneity, a random effects model was used for assessment (Figure 2).

## Flexural strength:

**Figure 3:**
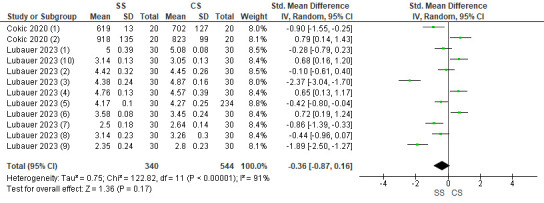
Forest plot for flexural strength

Two studies were included in the assessment. The pooled value obtained was -0.36[-0.87, 0.16] indicating that the flexural strength values were less with speed sintered zirconia as compared to conventionally sintered. Overall, the results were not statistically significant (p>0.05), with 91% heterogeneity. Due to high heterogeneity, a random effects model was used for assessment (Figure 3).

## Youngs Modulus:

**Figure 4:**
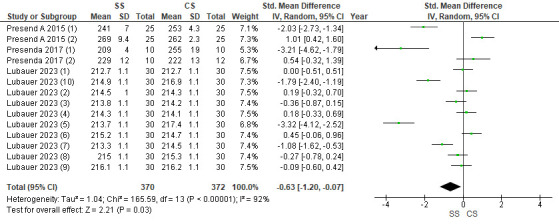
Forest plot for Young's modulus

Three studies were included in the assessment. The pooled value obtained was -0.63[-1.20, -0.07] indicating that the Young's modulus values were less with speed sintered zirconia as compared to conventionally sintered. Overall, the results were statistically significant (p<0.05), with 92% heterogeneity. Due to high heterogeneity, a random effects model was used for assessment (Figure 4).

## Hardness:

**Figure 5:**
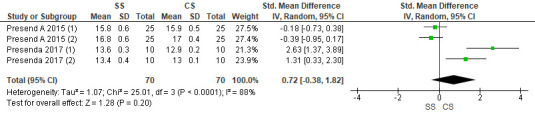
Forest plot for Hardness

Two studies were included in the assessment. The pooled value obtained was 0.72[-0.38, 1.82] indicating that the hardness values were greater with speed sintered zirconia as compared to conventionally sintered. Overall, the results were not statistically significant (p>0.05), with 88% heterogeneity. Due to high heterogeneity, random effects model was used for assessment (Figure 5).

## Discussion:

The present systematic review evaluated the influence of microwave sintering and conventional sintering on the mechanical properties of yttria-stabilized tetragonal zirconia polycrystalline (Y-TZP) material. Y-TZP demonstrates distinctive polymorphic properties, including monoclinic, tetragonal and cubic phases. This polymorphism imparts remarkable mechanical properties to zirconia, such as resistance to crack propagation and impressive fracture toughness [29]. The transformation toughening mechanism, associated with reversible phase transformations, contributes to zirconia's exceptional strength and durability. Yttrium oxide is added to pure zirconia to stabilize the tetragonal phase at room temperature and reduce volume expansion [18]. Conventional sintering, a well-established method uses electrical-resistance or gas-fired furnaces to heat zirconia powder gradually, typically over extended periods [24]. This traditional method, involving controlled temperature ramping, dwell times and cooling rates, has been a cornerstone in ceramic processing for many years. The extended sintering durations contribute to enhanced grain growth, influencing the final properties of the material. These furnaces use resistance heating elements such as molybdenum disilicide and silicon carbide and are equipped with gas control systems to create controlled atmospheres, preventing undesired reactions [13]. Microwave sintering, a relatively modern technique, utilizes microwave radiation for rapid heating within the zirconia powder. It involves electromagnetic waves to directly heat the material, resulting in rapid and uniform temperature distribution. This technique offers advantages such as reduced processing time, energy efficiency, and improved uniform heating [23- 25]. The rapid and uniform heating can lead to specific changes in the microstructure and properties of zirconia, resulting in enhanced mechanical properties. This review compared the performance of microwave sintering and conventional sintering from 2000 to 2023, focusing on relative density, Young's modulus, flexural strength, and hardness of zirconia [15-28]. The results suggest that some mechanical properties, such as hardness, are superior with microwave sintering, while properties like relative density, Young's modulus and flexural strength are greater with conventional sintering.

The findings indicate that conventional sintering results in higher relative density due to slower heating and longer dwell times, facilitating greater particle rearrangement, sintering neck formation, and densification. This leads to a more compact zirconia with less porosity. However, the differences were not statistically significant (88% heterogeneity). Conventional sintering also yields higher values of flexural strength and Young's modulus due to controlled heating and longer sintering times, allowing for improved crystal growth and better inter particle bonding. The results for Young's modulus were statistically significant (92% heterogeneity). In contrast, microwave sintering enhances hardness through rapid and uniform heating, forming a fine-grained microstructure with minimized grain boundaries, reducing defects and increasing overall hardness. Microwave sintering also presents certain challenges, such as sluggish grain growth during the final stage of sintering and slightly reduced fracture toughness due to shorter dwelling times (10-15 minutes). However, this technique is significantly faster than conventional sintering, saving time and energy in producing zirconia materials with adequate mechanical properties [24, 26]. Microwave sintering results in increased hardness by increasing the density (above 98%) of zirconia particles compared to conventional sintering. The field of zirconia sintering is marked by continuous innovation and refinement [15-21]. Recent advancements in both microwave and conventional techniques underscore the commitment to pushing the boundaries of what can be achieved with this versatile ceramic material [28, 29]. Researchers weigh various technical parameters and specific application requirements when selecting the most appropriate sintering technique for a given application in fields such as dentistry and advanced ceramics manufacturing. As researchers delve deeper into the intricacies of the sintering process, the future holds the promise of even more tailored and efficient methods for realizing the full potential of zirconia across a spectrum of applications.

## Conclusion:

The present systematic review highlights the distinct advantages and limitations of both microwave and conventional sintering techniques for yttria-stabilized zirconia (Y-TZP). While microwave sintering offers superior hardness due to its rapid and uniform heating process, conventional sintering yields higher relative density, Young's modulus, and flexural strength through controlled, extended heating. The choice between these methods should be guided by the specific mechanical property requirements of the intended application, with microwave sintering proving beneficial for energy efficiency and expedited processing, and conventional sintering ensuring optimal material density and strength. Future advancements in sintering technology are anticipated to further enhance the performance and application range of zirconia ceramics, promoting innovation in fields such as dentistry and advanced material manufacturing.

## Figures and Tables

**Table 1:** Characteristics of included studies

**Study ID**	**Place of study**	**Sample size**	**Intervention**	**Comparison**	**Material**	**Cycle**		**Outcomes measured**	**Authors conclusions**
		**IG/CG**				**Speed sintering**	**Conventional**		
Ai 2015	China	-	two step microwave sintering, one step microwave sintering	two step conventional sintering		900°C, 30min + 1350°C 30min	900°C 2h + 1350°C, 2h	density, hardness, fracture toughness, bending strength	No significant variation in density and mechanical properties between one-step and two-step microwave sintering. Microwave sintering superior to conventional sintering.
Presenda 2015	Spain	10/10	Microwave sintering	conventional sintering	LAVA	1200°C 10min + 1300°C 10min	1300°C 120min + 1400°C 120min	density, youngs modulus	Microwave sintering enhances mechanical properties, reduces time and energy consumption.
					VITA	1200°C 10min + 1300°C 10min	1300°C 120min + 1400°C 120min		
					TOSOH	1200°C 10min + 1300°C 10min	1300°C 120min + 1400°C 120min		
Presenda 2015 A	Spain	25/25	one step microwave sintering	one step conventional sintering	LAVA	1200°C 10min	1400°C 2h	relative density, hardness, young's modulus, surface topography	Significant influence on microstructure and hydrothermal degradation susceptibility.
					TOSOH (LAB)	1200°C 10min	1400°C 2h		
Presenda 2017	Spain	10-Oct	one step microwave sintering	one step conventional sintering	LAVA	1200°C 10min	1400°C 2h	relative density, hardness, young's modulus	Comparable wear resistance with lower sintering temperatures and shorter processing times.
					TOSOH (LAB)	1200°C 10min	1400°C 2h		
Presenda 2017 A	Spain	05-May	microwave sintering at 2 different temperatures - 1200C and 1300C	one step conventional sintering	10ZTA	1300°C 10min + 1400°C 10mon	1400°C 120min	density, hardness, fracture toughness	Reduces processing times and temperatures, increases resistance to LTD.
					5ZTA	1300°C 10min + 1400°C 10mon	1400°C 120min		
					5ATZ	1300°C 10min + 1400°C 10mon	1400°C 120min		
					3Y-TZP	1300°C 10min + 1400°C 10mon	1400°C 120min		
Kaizer 2017	USA	10-Oct	speed sintering	conventional sintering	inCoris TZI	Heating at 99 °C/min to 1100 °C, then at 50 °C/min to 1510 °C, dwelling for 30 min, followed by cooling at 99 °C/min down to 800 °C dwelling for 5 min before removing from the furnace. Total sintering time 60 min	Heating at 25 °C/min to 800 °C, then at 15 °C/min to 1510 °C, dwelling for 120 min, followed by cooling at 30 °C/min down to 200 °C before removing from the furnace. Total sintering time 4 h.	wear depth, wear volume, optical properties	Fast sintering improves microstructural, physical, and wear properties, but poorer antagonist wear.
Kauling 2019	Germany	48 24/24	speed sintering	conventional sintering	cerec zirconia medi	N/A	N/A	fit and fracture strength	Speed-sintered FPDs show equal/better fit and fracture load than conventional sintering.
Ozturk 2019	Turkey	340 16 groups n=10	speed sintering	sintered according to manufacturer's instructions	inCoris TZI, Upcera	Temperature 1400-1600°C, holding time - 30-240 min	According to manufacturer's instructions	surface roughness, flexural strength	No effect on surface phase transformation, roughness, and strength.
Moratal 2021	Spain	06-Jun	Microwave sintering at 2 different temperatures - 1200C and 1300C	conventional sintering	NK00	1. 1200°C, 10min 2. 1300°C, 10min	1400°C, 60 min	relative density,	Microwave technology increases sintering activity due to dielectric properties.
					NK04				
					NK08				
					NK10				
Cokic 2020	Belgium	20/20	speed sintered 1. Katana STML 2. CEREC zirconia	conventional sintered 1. Katana STML 2. inCoris TZI	Katana STML, inCoris TZI, CEREC zirconia	Total thermal cycle/sintering	Total sintering time 6.8h, dwell time 2h at 1550°C	density, flexural strength	Speed sintering suitable for clinical use but requires translucency and reliability improvements.
						time/dwell temperature: 30 min/16 min/1560 °C			
Yang 2020	China	15/15	rapid sintering	conventional sintering	Corpan zirconia system, Cercon HT, Cercon XT	Corpan: 50-1100°C, dwell time 30min Cercon: 70-1540°C, dwell time 35min	Corpan: 10-950°C, dwell time 90min Cercon: 22-880°C, dwell time 130min	flexural strength	Rapid sintering affects optical properties depending on the material.
Albayrak 2023	Turkey	40 20/20	speed sintering	conventional sintering	monolithic zirconia IPS emax CAD MO IPS emax CAD MT Lava Plus Lava Esthetic Cercon ht cercon xt Katana ML Katana STML Prettau Prettau Anterior	Total time 105min, 1515°C, dwell time 30min	Time: 7hrs, 1500°C, dwell time 120min	translucency values, opalescence, fluoroscence	Speed sintering increases translucency but causes minor changes in chemical composition.
Lubauer 2023	Germany	30/30	speed sintering	conventional sintering		Max temp:1500-1600°C dwell time: 120-145 mins	Max temp:1540°C dwell time: 35 mins	flexural strength, youngs modulus	No significant compromise in mechanical properties with speed sintering.
Rezeika 2023	Egypt	45 15/15/15	speed sintering according to manfacturer instructions	conventional sintering	inCoris TZI, IPS e-max	According to manufacturer's instructions	According to manufacturer's instructions	flexural strength, translucency parameter	Zirconia suitable for chairside use in non-aesthetic zones with superspeed sintering. Longer sintering required for high translucency restorations

**Table 2:** Quality assessment according to MINORS tool

**Study ID**	**Sample size**	**Random**	**Sintering**	**Sample preparation**	**Statistical analysis**	**Measuring procedures**	**Operator**	**Total**	**Risk of bias**
Ai 2015	2	2	0	1	0	0	2	7	Medium
Presenda 2015	0	1	0	0	0	0	1	2	Low
Presenda 2015 A	0	2	0	0	0	0	1	3	Low
Presenda 2017	0	2	0	0	0	0	1	3	Low
Presenda 2017 A	0	2	0	0	0	0	1	3	Low
Kaizer 2017	0	1	0	0	0	0	1	2	Low
Kauling 2019	0	2	0	0	0	0	1	3	Low
Ozturk 2019	0	2	0	1	0	0	1	4	Low
Cokic 2020	0	2	0	0	0	0	1	3	Low
Yang 2020	0	2	0	0	0	0	1	3	Low
Moratal 2021	0	2	0	0	0	0	1	3	Low
Albayrak 2023	0	2	0	0	0	0	1	3	Low
Lubauer 2023	0	2	0	0	0	0	1	3	Low
Rezeika 2023	0	1	0	0	0	0	1	2	Low
